# “You lose the person; they're still there but you don't recognize them”: A qualitative study examining the consequences of conspiracy beliefs for romantic partners

**DOI:** 10.1111/bjso.70033

**Published:** 2025-12-12

**Authors:** Lea C. Kamitz, Ricky Green, Cassidy Rowden, Daniel Toribio‐Flórez, Mikey Biddlestone, Karen M. Douglas

**Affiliations:** ^1^ School of Psychology University of Kent Canterbury UK; ^2^ Present address: International Policing and Public Protection Research Institute Anglia Ruskin University Chelmsford UK

**Keywords:** consequences, conspiracy beliefs, conspiracy theories, qualitative research, relationships, thematic analysis

## Abstract

This study examined how conspiracy beliefs influence romantic relationships. We conducted semi‐structured interviews with 17 partners (or ex‐partners) of conspiracy believers, asking questions about their experiences in their relationships. A thematic analysis generated several key themes. Specifically, participants described how their partner's beliefs led to *relational strain* in the form of conflict and communication breakdown, which was tied to the psychosocial death of the partner and the relationship. There were several *negative effects on participants*, as their partner's beliefs not only affected their relationship dynamic but also took a psychological and physical toll on the participants themselves. *Sense‐making* became important as participants tried to understand their partner's beliefs. Ultimately, most relationships deteriorated, and many ended, though some remained intact due to financial or emotional barriers. During this process, participants reported *seeking support* and started to *navigate endings* in the case of relationship dissolution. These findings extend current knowledge on the consequences of conspiracy theories for interpersonal relationships, suggesting that they pose significant barriers to successful romantic attachments.

## INTRODUCTION

In recent years, conspiracy theories have received increasing attention from researchers, not only for their prevalence (see Duffy & Dacombe, 2023; Orth, 2023) but also for their association with a wide range of negative societal consequences (Douglas, 2021a; Douglas & Sutton, 2025). Numerous anecdotal reports suggest that the consequences of conspiracy theories extend beyond public health and politics and that their impact on interpersonal relationships is becoming increasingly evident. Stories of family tensions, friendship breakdowns and strained romantic relationships are becoming commonplace (e.g., Howe, 2024; Nagesh, 2021; Perry, 2024). Although recent research highlights some of the detrimental consequences of conspiracy theories for interpersonal relationships (e.g., Toribio‐Flórez et al., 2024; for a review, see Toribio‐Flórez et al., 2023), this subject remains largely understudied. Specifically, very little is known about the lived experiences of individuals whose romantic relationships have been affected when the other partner believes in conspiracy theories. This study aimed to address this gap by using qualitative methods to explore how romantic relationships are shaped and strained by conspiracy beliefs.

### The appeal and consequences of conspiracy theories

Conspiracy theories are beliefs that significant events have been secretly orchestrated by two or more people for nefarious purposes (Douglas & Sutton, 2023). Popular examples include claims that COVID‐19 was either a government hoax or a secret bioweapon (Douglas, 2021b), that pharmaceutical companies hide information about the safety and efficacy of immunization programs (Jolley & Douglas, 2014a), and that the ‘deep state’ influences election outcomes (Dover, 2023). Research suggests that people are drawn to conspiracy theories in an attempt to satisfy three fundamental psychological needs (Douglas et al., 2017; for a meta‐analysis, see Biddlestone et al., 2025): existential needs (to feel safe, secure and in control), epistemic needs (to gain certainty and understand the environment) and social needs (to enhance self‐esteem and foster positive perceptions of the groups they belong to). Rather than satisfying these needs, however, research suggests they may frustrate them even further (Douglas et al., 2017). For example, conspiracy beliefs tend to increase feelings of anxiety and uncertainty over time rather than alleviate these feelings (Liekefett et al., 2021). Other research has found that whilst increased feelings of meaning of life are associated with increased conspiracy beliefs, such beliefs lead to decreased feelings of control and uncertainty over time (Albath et al., 2024).

Conspiracy theories therefore appear to do more harm than good (Douglas, 2021a; Douglas & Sutton, 2025; Jolley et al., 2022), and their societal consequences also appear to be wide‐ranging. For example, meta‐analytic research found that climate change conspiracy beliefs are linked to distrust around the science behind climate change, lower support for pro‐climate policies and lower intentions to carry out behaviours aimed at mitigating the impact of climate change (i.e., recycling; Biddlestone et al., 2022). People who believed in COVID‐19 conspiracy theories were less likely to engage in virus‐mitigating behaviours, such as washing hands and social distancing (Biddlestone et al., 2020) and were less likely to take a COVID‐19 vaccine, opting instead for alternative cures, such as hydroxychloroquine (Bertin et al., 2020). Furthermore, people who believed that 5G masts were being installed to spread COVID‐19 were more likely to justify the use of violence against the engineers involved in their construction (Jolley & Paterson, 2020). Similarly, political conspiracy theories, such as those about election fraud or ‘deep state’ actors, have been linked to decreased normative political actions (i.e., voting; Imhoff et al., 2020; Jolley & Douglas, 2014b) and increased acceptance of political violence (Vegetti & Littvay, 2021). For example, conspiracy beliefs surrounding election fraud have been argued to be a key motivator for the January 6th Capitol riots (Enders et al., 2024).

Recently, researchers have turned their attention to the consequences of conspiracy beliefs for individuals. For example, research has found that people expect to be negatively evaluated and socially excluded if they were to endorse (vs. criticize) conspiracy theories about the Charlie Hebdo terrorist attack (Lantian et al., 2018), suggesting that conspiracy beliefs are perceived to be a source of stigma. Furthermore, conspiracy believers are often perceived as gullible, paranoid and lacking intelligence (Klein et al., 2015). People are also less likely to use the label ‘conspiracy theory’ when discussing narratives that they personally believe in (Douglas et al., 2022), while strategically using the term to discredit or trivialize others' explanations of events (Martin, 2020). More recent research has examined the reputational consequences for professionals, such as politicians and scientists, who endorse (vs. refute) conspiracy theories, finding that people form negative impressions (i.e., lower impressions of intelligence and trustworthiness) and are less willing to support them or follow their advice (e.g., Green, Toribio‐Flórez, & Douglas, 2023; Green, Toribio‐Flórez, Douglas, Brunkow, & Sutton, 2023; see also Cao et al., 2024). Gundersen et al. (2025) also found that people are aware of conspiracy believers' higher willingness to engage in conspiratorial behaviours (see Alper et al., 2024; Douglas & Sutton, 2011). Taken together, these findings suggest that there are social consequences for individuals who believe in and share conspiracy theories.

### Interpersonal consequences of conspiracy beliefs

Conspiracy beliefs also appear to have consequences for people's interpersonal relationships. Indeed, numerous media reports highlight how conspiracy beliefs can strain interpersonal relationships, including friendships and romantic and family relationships. For example, one such story reported that QAnon conspiracy beliefs led to a mother accusing her daughter of siding with evil forces, resulting in their estrangement (Nagesh, 2021). Similarly, two additional news reports highlighted that conspiracy theories during the COVID‐19 pandemic contributed to the breakdown of a 30‐year friendship (Perry, 2024) and nearly ended a marriage before one partner began to question their beliefs (Howe, 2024). More generally, research has shown that irrational beliefs negatively influence romantic relationships (e.g., Hamamci, 2005; Möller et al., 2001; Möller & De Beer, 1998). However, whilst such beliefs tend to concern the relationship itself, conspiracy beliefs revolve around external socio‐political issues that strain the relationship indirectly.

Building on this evidence that belief systems can affect relational functioning, recent research has begun to support the concerns surrounding the relational consequences of conspiracy theories. For example, Okdie et al. (2023) found that if a hypothetical family member were to endorse a conspiracy theory that was perceived as harmful (i.e., Climate change is a hoax) versus harmless (i.e., The Earth is flat), people were less likely to tolerate their belief and were more likely to confront them about it. Moskalenko et al. (2023) also found that people's relationships worsened due to a loved one's belief in QAnon conspiracy theories. Toribio‐Flórez et al. (2023) reviewed potential mechanisms through which conspiracy beliefs might damage interpersonal relationships, such as attitudinal distancing (i.e., non‐believers perceive a change in conspiracy believers' attitudes about certain topics that creates distance), stigmatization (i.e., non‐believers view conspiracy believers as gullible, paranoid, or unintelligent due to their beliefs) and norm violations (i.e., non‐believers observe behaviours such as rejecting COVID‐19 vaccines and endangering public health). Toribio‐Flórez et al. (2024) found that people who do not show a strong endorsement of conspiracy theories reported lower relationship satisfaction with others whom they perceived to believe in conspiracy theories, partially due to perceptions of attitudinal distancing. These findings suggest that conspiracy beliefs have the potential to strain relationships, eroding relationship satisfaction for non‐believers.

Qualitative research has examined these effects further. Waltman (2023) analyzed quotes from media accounts of QAnon's impact on interpersonal relationships and found, through thematic analysis, that QAnon drives a wedge into these relationships. Also, Mastroni and Mooney (2024) conducted in‐depth interviews with people who had QAnon‐affiliated loved ones (friends, family members and partners), uncovering four central themes: ‘Malignant Q’, which captures the perception of QAnon as a harmful and divisive influence; ‘Distance’, referring to both emotional and physical disconnection; ‘Qonflict’, highlighting instances of arguments and boundary violations; and ‘Attempts at Healing’, which reflects participants' strategies to salvage or cope with their relationships. These findings provide a rich understanding of the relational toll of QAnon conspiracy beliefs.

Although researchers have started to examine the interpersonal consequences of conspiracy beliefs, qualitative research is limited to the specific case of QAnon conspiracy beliefs (Mastroni & Mooney, 2024; Waltman, 2023). While QAnon conspiracy beliefs are a particularly extreme and US‐centric conspiracy movement (Miller, 2023), conspiracy beliefs in general are widespread and may have varying effects on relationships depending on their content and context. Moreover, existing qualitative research has focused on relationships generally (i.e., family, friends and significant others; Mastroni & Mooney, 2024), rather than focusing specifically on romantic relationships. Given the deep emotional (e.g., love, trust) and practical interdependence (e.g., shared finances and parenting responsibilities) in romantic relationships, these relationships may be especially vulnerable to strain when one partner believes in conspiracy theories. For example, disagreements may arise over medical decisions, such as vaccinating children or rejecting conventional medicine (Bertin et al., 2020; Jolley & Douglas, 2014a), and tensions may build if one partner rejects climate science and opposes environmentally conscious behaviours valued by the other (Biddlestone et al., 2022). There is therefore a need to examine how conspiracy beliefs in general shape romantic relationships, beyond the specific case of QAnon.

In the present research, we conducted semi‐structured interviews with 17 people who are currently, or who have been, in relationships with conspiracy believers. We then conducted an exploratory qualitative analysis using inductive thematic methods (Braun & Clarke, 2006). Our study aimed to uncover how conspiracy beliefs influence and shape interpersonal relationships, particularly within the context of romantic relationships. Given the lack of prior research in this area, our approach is deliberately exploratory, with no pre‐specified hypotheses guiding the analysis. Inductive thematic analysis offers a flexible and data‐driven approach to identifying patterns and themes within qualitative data (Braun & Clarke, 2006). In line with this approach, themes were developed through an active and interpretative process, shaped by our engagement with the data. This allowed us to construct a rich understanding of participants' experiences by developing themes through close, interpretative engagement with participants' accounts, without being constrained by pre‐existing frameworks (Clark & Braun, 2021; Nowell et al., 2017).

## METHOD

### Ethics

Ethics approval was obtained from the University of Kent School of Psychology Ethics Committee (ID: 202316925535658613). Prior to the interviews, informed consent was given verbally by each participant. Following the interviews, participants were given a full verbal debriefing. Participant numbers are used in the reporting of this research to protect participants' identities, and all identifiable information has been removed from the research output.

### Participants

We recruited 17 participants (10 female and 7 male), with ages ranging from 28 to 66 years (*M* = 45.12, *SD* = 10.30). Most participants were white (70.6%; *n* = 12), 2 self‐described as Latina/Hispanic (11.8%), and one participant each described themselves as Asian (Indian), Black, or from a mixed ethnic background (5.9%). At the time of the interviews and during their relationships, 6 participants resided in the UK (35.3%), 6 in the USA (35.3%), and one participant each resided in India, Canada, the Netherlands and Australia, respectively (5.9%). One participant lived in the UK during their relationship with someone who believed in conspiracy theories but had moved to the USA at the time of the interview.

#### Relationship characteristics

Eleven of the participants' partners and former partners who believed in conspiracy theories were male and six were female. One participant was in a same‐sex relationship and all other participants were in heterosexual relationships. The total duration of the participants' relationships, at the time of interview, ranged from 1.5 to 31 years (*M* = 12.53, *SD* = 8.86). At the time of interview, most participants (76.5%, *n* = 13) were not in the relationship in question anymore. Recent statistics suggest that around 42% of marriages in England and Wales eventually end in divorce, with 8.9 divorces per 1000 married people each year (Office for National Statistics, 2025). Relationship dissolution is therefore common in the general population. However, of the participants in the current research, the majority (*n* = 11, 84.6% of those whose relationship ended, 64.7% of the entire sample) stated that their relationship ended as a direct result of their partner's involvement with conspiracy theories. Participants' partners endorsed conspiracy theories across a range of themes, including public health and medicine (e.g., COVID‐19 as a hoax or bioweapon, anti‐vaccine beliefs, denial of AIDS), politics and government (e.g., QAnon, beliefs in a shadow government often linked to antisemitic narratives), science and technology (e.g., climate change denial, 5G, 15‐minute cities), gender and society (e.g., manosphere‐related beliefs) and governmental cover‐ups (e.g., 9/11 as an inside job, government concealment of extraterrestrial life). In our sample, all partners endorsed multiple conspiracy theories.

### Participant recruitment

Eleven participants contacted the researchers after seeing the researchers' call for participants on the Reddit forum r/QAnonCasualties. This forum aimed to provide “support, resources and a place to vent” for those whose friends or family members have been “taken in” by QAnon, specifically (r/QAnonCasualties, n.d.). Whilst the forum often focuses on QAnon, the community rules encourage posts related to interactions with any “Q/Adjacent” people in the poster's social circle. As such, many submissions to the forum discuss users' relationships with individuals believing in a wide range of conspiracy theories. A further 4 participants contacted the researchers in response to the call for participants published on the researchers' profiles on the social media website X. The remaining two participants were contacted by the researchers after these participants had previously emailed the researchers with questions relating to their experiences of being in a relationship with someone who believes in conspiracy theories. After contacting the researchers via email, prospective participants completed a survey to assess their eligibility and record their demographic characteristics. During this survey they also generated a unique code which ensured that we could process their data without compromising their anonymity. We made appointments for Teams audio or video calls – depending on each participant's preference – with eligible participants via email. Upon interview completion, all participants received a reimbursement of £10 (or equivalent) in the form of an Amazon voucher.

### Data collection

All interviews were conducted by the first author. During the interviews, participants were asked to detail potentially important aspects of their relationship, including the beginning, progression and, where applicable, the end of their relationship, and the impact their partner's belief in conspiracy theories had not only on the relationship but also on the participant themselves. Here, the focus was on the participants' own thoughts and feelings. A semi‐structured interview schedule was used as a guideline to ensure important aspects of the participants' experiences were covered while allowing the participants to lead the interview and expand on issues they deemed important. Due to this participant‐led approach and the uniqueness of each participant's experience, the interviews varied in duration from 22 to 76 minutes (*M* = 46.24 minutes, *SD* = 17.42 minutes). Interviews were recorded with the participants' knowledge and consent and were transcribed verbatim by the Microsoft Teams live transcription function. All transcripts were subsequently manually checked against the audio recordings.

### Analysis

Analysis of the interview transcripts was conducted by the first author using inductive thematic analysis, according to Braun and Clarke (2006). The first step was familiarization with the data by reading and re‐reading each transcript carefully while noting down initial thoughts and patterns. The transcriber then generated initial codes by going through the data systematically. For example, the following excerpt, from the interview with Participant 12, was coded as “Losing and not recognising partner”:

You lose the person, they're still there but you don't recognise them.

Coding was, wherever possible, done in gerunds which, in contrast to coding in topics and themes, enables the researcher to study processes, actions and implicit connections (Charmaz, 2014). Subsequently, the first author began grouping codes into potential themes and subthemes. For instance, the excerpt above was assigned the subtheme *Psychosocial Death of Partner and Relationship* and eventually grouped into the theme *Relational Strain*. Following this, each theme was reviewed before producing definitions and names for each theme. Here, the researcher followed an iterative approach, meaning that they generated and refined codes and themes throughout.

Consistent with Braun and Clarke's (2021) distinction between different conceptualizations of themes, our analysis produced what Braun and Clarke refer to as ‘themes‐as‐topics’: organized patterns capturing important aspects of participants' relationship experiences, rather than shared‐meaning themes. Given the exploratory nature of the study, a topic‐centred structure allowed us to represent both the diversity and complexity of participants' accounts.

### Reflexivity

Any potential biases arising from prior assumptions about the topic were minimized by the fact that the first author, who conducted all interviews and analyses, had no previous contact or research experience with partners of those who believe in conspiracy theories or conspiracy theorists themselves. Throughout the interview process, analysis and write‐up of the findings, the first author additionally engaged in reflexivity by reflecting on and challenging arising biases and emotions towards the topic and noting these reflections down in a research journal. For instance, after the first two interviews conducted, the first author noted that she felt some sense of inadequacy after being repeatedly asked by participants whether she knew about certain prominent figures or movements within the conspiracy sphere. As an interviewer, the first author retrospectively recognized that, in the moment, she was worried that she seemed ill‐prepared or incompetent. After reflecting on this experience in her diary, she noted that she was not supposed to be the “expert” in these interviews and that this role firmly belonged to the interviewee, giving her confidence to be candid about her naivety regarding the topic of conspiracy theories. Ultimately, reflecting on these interactions allowed the first author to realize just how knowledgeable each participant had become about the conspiracy sphere because of the developments in their relationship, partially leading to the development of the subtheme *Becoming Experts*.

In addition, the first author acknowledges that she does not personally believe in conspiracy theories and considers them to be potentially harmful to individuals and society. Her views on science and politics aligned with those of many participants, being pro‐science and politically liberal, which may have influenced her interpretive lens. Whilst these shared values contributed to strong rapport and mutual trust with interviewees, they also presented the risk of overidentification with the participants' perspectives. The first author actively reflected on this alignment throughout the research process, particularly considering how it might have shaped her interpretation of participants' accounts. These reflections were revisited in analytic memos and journaling during the analytical process to ensure that participants' narratives were represented fairly.

## FINDINGS

**FIGURE 1:**
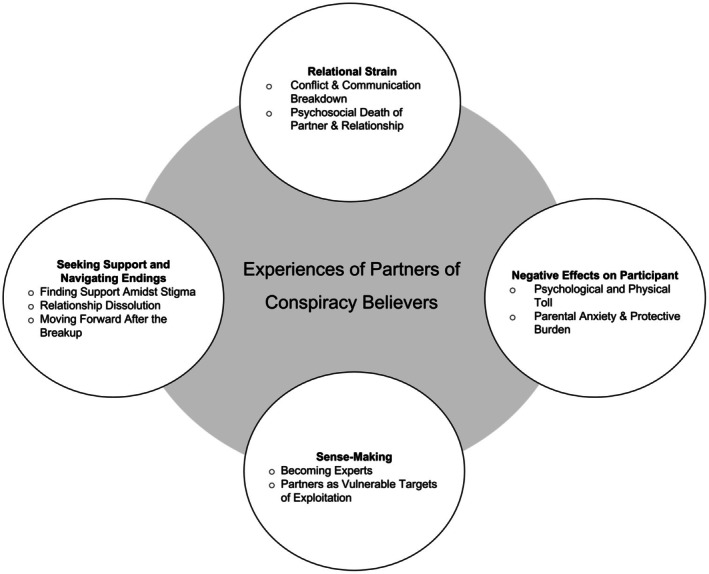
Themes and subthemes. Themes are in bold. Subthemes are in bullet points. Themes are presented clockwise as they appear in‐text, beginning with *Relational Strain*.

The analysis generated themes that capture the complex experiences of individuals whose partners are conspiracy believers. These themes highlight emotional, relational and practical challenges faced, as well as participants' coping and sense‐making strategies. Figure 1 summarizes the themes and subthemes, which will be discussed below.

### Relational strain

For most participants, their partner's conspiracy belief significantly shifted and disrupted their relationship, leading to conflict, communication breakdown by trying to avoid such conflict, as well as a growing sense that their partner – and the relationship itself – had become unrecognizable.

#### Conflict and communication breakdown

In the early stages, participants were often hopeful that they would be able to simply debunk their partner's views by presenting them with factual information and engaging in debate. As this proved ineffective, participants sometimes viewed themselves as naive for thinking that a logical approach would soften their partner's views, and expressed the belief that attempts to reason with conspiracy believers are pointless, coming to the realization that confronting their partner's views often worsened conflict (e.g., “I quickly realised it was completely pointless because no matter what you showed him, if it didn't match what he wanted to believe, he would just say that's mainstream media or that's the deep state paying them off or whatever.”; Participant 3). Simultaneously, the participants' partners also repeatedly tried to convince the participants of their beliefs. However, the ineffectiveness of ‘rational’ debate often resulted in a deadlock, leading to emotional frustration and a lack of resolution.

As conspiracy theories became more central to their partners' worldview and increasingly dominated the relationship, arguments became more frequent and intense. This was the case even for participants whose relationships had been relatively conflict‐free previously. Over time, these arguments became more personal and emotional, and occasionally even aggressive. Participants described being personally targeted and insulted for their lack of belief, often being labelled “brainwashed”, “a sheep” or even an “enemy” (e.g., “He would accuse me of being brainwashed and a sheep.”; Participant 6). The intensity of these conflicts and the resulting emotionality were often perceived to only entrench both partners' stances and led to emotional exhaustion, alienation and enduring relational strain.

As a result of these escalating and futile confrontations, many participants began to avoid conversations about conspiracy theories, either unilaterally or based on a mutual understanding between themselves and their partner. Sometimes, this led to a temporary improvement in the relationship dynamic. However, participants found it increasingly difficult to avoid conspiracy theories as a conversational topic due to the omnipresence of their partner's beliefs in daily conversations. Participants described being unable to connect to their partner anymore because of a lack of communication (e.g., “We've had a fairly sort of peaceful last few weeks because we basically agreed not to talk about any of that stuff at all, which is quite difficult because there's unbelievably a conspiracy [theory] related to practically everything.”; Participant 8). This pattern of futile engagement, personal attacks and avoidance was reflective of a communication breakdown that participants found emotionally depleting and unsustainable.

#### Psychosocial death of partner and relationship

In extreme cases, the changes observed in their partner included abusive and dangerous behaviours. As their partner's conspiracy beliefs deepened, participants felt emotionally disconnected and reported major behavioural and psychological changes in their partner. They described that, since believing in conspiracy theories, conspiracy content was all their partner talked about and spent time researching – specifically on social media platforms such as YouTube and Facebook, on the dark web, or through podcasts. Because of this, their partner also often became isolated and withdrew from family and friends who did not share their beliefs. Participants described the experience as the “death” of the person they once knew, noting that engaging with conspiracy content significantly changed their partner's character, making them angry, agitated, mean, aggressive and hateful, but also fearful and withdrawn (e.g., “He became this very angry, depressed, unhappy person, you know, who took it out on people around him.”; Participant 9). Notably, participants described their partner as a completely different person, referring to them as a stranger, saying that they do not know their partner, questioning whether their partner was who they thought they were, or reporting feeling like their partner had been replaced (e.g., “She would say to me ‘I'm still the same person.’ But then are you the same person? I mean, what makes up a person is your beliefs, desires, your hopes… So, if you suddenly, like, have a sort of different view on everything that you ever thought of and believed before… You know, are you the same person?”; Participant 8). This, coupled with expressions referring to the “loss” of not only their partner but also their friend, and descriptions of only rarely seeing a “glimmer” of the “old person” evoke notions of grief and mourning which illustrate just how negatively impactful a partner's conspiracy belief can be. For participants with children, this also extended to co‐parenting, as they mourned the loss of the co‐parent they thought they had and the inability to provide their children with the parent they wanted for them. At the same time, participants reported a lack of empathy and social support from others during this grieving process (as further explored in *Finding Support Amidst Stigma*), leading to disenfranchised grief. Resignation to the fact that their partner would likely never change contributed to feelings of powerlessness as explored in *Psychological and Physical Toll*.

In some extreme cases, participants perceived their partner as having become dangerous for themselves and others. Here, participants reported fearing their partner because of threats or abusive behaviour (including physical violence), which were directly related to conspiracy beliefs. For example, one participant described the moment his partner found out that he, the participant, has received the COVID‐19 vaccine: “I got her son to come and take her away because she was hitting me, screaming, punching me. Because she thought that I would have given it, given the vaccine to her somehow.” (Participant 2). Two participants were threatened due to their work as healthcare professionals during the COVID‐19 pandemic (e.g., “It just really got to the point where I felt, I mean, truly threatened. He was saying, you know, ‘you're gonna suffer a fate far worse than regret. It's not gonna be me, but people are, people will have their way with you.”; Participant 16). When participants viewed their partner as dangerous, they often started gathering evidence or reporting behaviour to the police or extremism hotlines, motivated by concerns both for their personal safety and about the risk their partner may pose to others.

### Negative effects on participants

Most participants reported that their partners' conspiracy belief, and especially the escalating arguments, as described above, had detrimental consequences for participants' health and wellbeing. This was exacerbated by constant exposure to conspiracy content and the emotional labour of exhausting, but futile, debunking efforts, and the associated powerlessness felt by participants (e.g., “It was exhausting. I've never felt as tired in my life as I did around that time. It's hard. 2020 to 2021 was the worst year of my life.”; Participant 3). For those who had children with their partner, shielding them from their partner's beliefs became an additional burden.

#### Psychological and physical toll

Participants experienced a wide range of negative mental and physical health consequences stemming from their partners' conspiracy beliefs. Many reported heightened anxiety, chronic stress, depression, insomnia and coping behaviours such as increased alcohol consumption, which severely affected their overall wellbeing (e.g., “I'm a very anxious person to begin with and hearing him rant about this stuff made me even more anxious, and I drank a lot more because I couldn't sleep.”; Participant 6). Physical health issues related to prolonged stress, like thyroid problems, were also described alongside financial difficulties caused by diminished work focus. Participants also frequently experienced self‐doubt, disorientation and confusion. Their partners' unwavering certainty in conspiracy theories led them to question their own sanity (e.g., “Sometimes you can get so overwhelmed cause he seems so sure. And it messes with your head. You start thinking you're the crazy one.”; Participant 3). This sense of disorientation was compounded by feelings of helplessness. Despite early hopes that logical debate might change their partners' views, as described above, repeated failures to do so led participants to feel powerless. They came to believe that there was nothing they could do to change the situation as exiting conspiracy beliefs was ultimately a choice their partner had to make for themselves, which deepened emotional detachment and feelings of despair (e.g., “It's very hard when you really love someone and, you know, like I really wanted to help them. But there is no… There's nothing I could do unfortunately.”; Participant 14).

#### Parental anxiety and protective burden

Participants with children expressed concern about their children being exposed to harmful conspiracy beliefs through the child's other parent and reported that it was their aim to protect their children from this influence. Participants worried not only about ideological influences but also about serious consequences for their child's health. In terms of ideological influence, participants described that their partners shared their beliefs with their child and even actively involved them, for instance, by taking the child to rallies and introducing them to conspiracy influencers. To protect their child, participants reported trying to educate their children to evaluate evidence and noted that they were proud of their children for not believing in conspiracy theories but retaining their moderate political beliefs despite their partner's attempts to “convert them”.

One of the main parenting and child protection concerns, however, arose around medical care, especially vaccinations. Participants reported feeling that their partner was medically neglecting their child by not allowing the child to receive necessary vaccinations. In one case, this resulted in the participant's child requiring hospital treatment because of a preventable disease (“Our daughter obviously isn't vaccinated or anything. She's been hospitalised twice. The hospital literally said you've caused this 'cause she's not had the vaccine”; Participant 2). To protect their children, some participants secretly took their children to get vaccinated. Others' medical worries extended beyond vaccination: Participants worried that their partner might not allow their child to receive sufficient medical help in the case of an emergency. In some cases, fears around radicalization and medical neglect were a main reason for leaving the relationship to better protect their children. Overall, even though participants went to great lengths to protect their children, they still described feeling guilty about not providing their children with the parent they would have wanted them to have.

### Sense‐making

Due to the impact their partner's beliefs had on their relationships and themselves, and the lack of knowledge participants perceived themselves to have about the conspiracy sphere, participants gathered information in an attempt to act appropriately and make sense of the drastic changes in their lives. This not only included learning about conspiracy theories but also forming an understanding of the reasons for their partner's beliefs and the motives of those who promote them.

#### Becoming experts

Throughout each interview, it became apparent that participants knew far more about the conspiracy sphere than the average person. Participants described researching conspiracy theories and prominent conspiracy theorists across online sources and books to gain a better understanding of the issues facing their partner and their relationship. Others took part in conspiracy‐related academic research, engaged in online forums on the topic and accessed therapy to try to understand why their partner started engaging with conspiracy theories and became radicalized. Whilst many viewed this as a systematic approach to addressing specific issues within their relationship, such as “staying one step ahead” in debates or learning how to engage with someone who believes in conspiracy theories, others described engaging with this behaviour predominantly as a way of coping with the change in their partner and the relationship (“For me, being able to kind of analyse the phenomenon and relating it back to how I can see her believe something is…Over time is kind of my way of coping. You know, logically it makes no sense, but if I can try to understand it in some way I can come to terms with how it was and how it blew up”; Participant 7). For some participants, this interest continued even after their relationship ended: Participants reported wanting to support others in similar situations, and one participant even began writing academic papers on the topic of conspiracy theories and made this work their full‐time job.

#### Partners as vulnerable targets of exploitation

Many participants likened their partner's conspiracy belief to addiction, mental illness, or cult indoctrination, seeing these as powerful forces that trapped their partner and brainwashed them. This was often tied to a perceived lack of agency, with partners viewed as passive victims preyed upon by others rather than willing participants (e.g., “We are literally trying to save our loved ones from these cult groups”; Participant 15). Participants especially tended to view their partners as vulnerable if they had pre‐existing mental health issues, substance abuse issues, trauma, or ultra‐religious upbringings that discouraged critical thought. Some were unsure whether their partner's conspiracy beliefs reflected political ideology or symptoms of poor mental health, and this uncertainty often led them to stay despite negative effects, assuming a duty of care (e.g., “You think how much of this is his choice and how much of this is a pathology? I think one of the reasons I hung in for as long as I did was 'cause I thought that he needed help and I had to get him to a place where he would be accepting of that help. But, of course, that never happened.”; Participant 10).

In line with this perception of reduced agency, participants perceived the conspiracy sphere and so‐called conspiracy influencers as exploitative agents who prey on dissatisfied and vulnerable people (e.g., “A lot of conspiracy theorists, like on the high level, they prey on people who think that life hasn't become what they want”; Participant 2). Whilst some believed influencers aimed to radicalize others for ideological purposes, participants most commonly saw them doing so for personal gain, such as financial profit.

### Seeking support and navigating endings

Faced with the challenges caused by their partners' conspiracy beliefs, participants often struggled to cope alone and sought emotional and practical support from their social networks and professionals. Whilst some found comfort in confiding in others, many encountered stigma, isolation, or judgement. Over time, the strain on relationships frequently led to deterioration and, for many, eventual relationship dissolution. Although some experienced relief and healing after ending their relationship, many faced significant emotional and practical barriers in leaving, and others remained uncertain about the future of their partnership. This theme explores how participants sought support, made decisions about ending their relationships and navigated life afterwards.

#### Finding support amidst stigma

Most participants confided in at least one other person about their partner's conspiracy belief, such as a friend, family member, colleague, or a member of an online support group. For many, spaces like the QAnon Casualties forum on Reddit provided much‐needed community and understanding (e.g., “I think one thing I found really helpful was finding the QAnon casualties forum [on Reddit]. Because you feel like you're the only one in that situation. Um, and it's bonkers, and no one else gets it, and anyone you talk to, they just don't get it. Whereas I found there's all these people in the same situation as me, and that has been so helpful.”; Participant 10). Participants chose carefully whom to confide in, often restricting this to emotionally close contacts or those already aware of the situation, due to fears of judgement or misunderstanding.

However, sharing this information was not without risks. Many participants experienced isolation and social stigma, feeling unfairly held accountable for their partner's behaviours, such as controversial social media posts or decisions like vaccine refusal, which led to feelings of shame and misunderstanding (e.g., “My husband's sister is an opioid addict. With addiction, at least people feel sorry for you. That's the thing that's hard about this. You usually just get shame and criticism”; Participant 12). This stigma was compounded by others minimizing the seriousness of conspiracy beliefs, further pushing participants towards social withdrawal. Some chose to hide or downplay their partner's beliefs to avoid judgement or to preserve the hope that their partner might “come back” from conspiracy belief.

Many participants sought professional support to navigate the emotional and practical difficulties in their relationships. Some accessed therapy or counselling independently, sometimes secretly, to develop coping strategies and gain advice. Others attended couples therapy, reflecting a determination to maintain their relationship despite the strain. Finding suitable professional help often required effort, and experiences varied. While some found validation and relief in speaking openly with a knowledgeable professional (e.g., “It's been quite useful, just getting out there. Getting it off your chest, having someone who's listening to you sort of go on about it.”; Participant 8), others encountered professionals who lacked understanding of conspiracy theories, dismissing participants' concerns or even sharing conspiratorial beliefs themselves. This often left participants feeling misunderstood and further isolated.

#### Relationship dissolution

Participants or their partners often reached a breaking point where they recognized that their relationship had become unsustainable, resulting in either party initiating a breakup or a mutual agreement to end the relationship. Common reasons included emotional detachment and loss of attraction due to their partner's conspiracy beliefs. Participants also described ending relationships because of anger and aggression they linked to their partner's engagement with conspiracy theories. The futility of trying to change their partner's beliefs, along with the resulting incompatibility of views and lifestyles, led to frequent arguments and a sense of living in two separate realities. For example, multiple participants reported breakups related to COVID‐19 vaccination disagreements (e.g., “When I got vaccinated, she said ‘That's it. I can't live with someone [who is vaccinated] because it's gonna kill you.’ She thought she could catch it off me.” (Participant 2)). In some cases, the partner's conspiracy beliefs exacerbated pre‐existing relationship issues, magnifying differences and contributing to the relationship's end. Support from family, friends and professionals was often critical in helping participants decide to leave. One participant described, “I mean, we did eight sessions [with a relationship counsellor] before it became very clear that this was not going to be viable, and that divorce was imminent” (Participant 7). External support systems also provided vital emotional and practical assistance during and after the breakup, such as a place to stay or childcare to enable participants to resume work.

Despite wanting to leave, some participants faced significant emotional and practical barriers that delayed or prevented them from ending their relationships. These included long relationship duration, concerns about children, financial dependence and lack of support, contrasting with those who benefited from external support. Some hesitated due to worries about their partner's mental health, reflecting the sense‐making processes where conspiracy belief was sometimes seen as related to psychiatric issues. Feelings of shame about having been in a “failed” relationship also played a role, particularly for those with prior relationship breakdowns. Finally, one participant highlighted immigration status as a barrier: “I couldn't leave because my residence immigration status was tied to my relationship with him.” (Participant 6).

#### Moving forward after the breakup

After the end of the relationship, many participants experienced relief, inner peace and emotional healing. For example, one participant shared, “I was sad, but I was mentally at peace after over a year of hell. Sleeping again… It was very sad but there was some peace in having decided this is over and I'm leaving.” (Participant 3). For most, healing involved distancing themselves from their ex‐partner and cutting all contact to avoid further exposure to their partner's conspiracy beliefs. However, some participants maintained contact if disengagement from the conspiracy sphere was possible or for co‐parenting.

For participants with children, co‐parenting with a conspiracy‐believing ex‐partner presented major challenges. They described balancing the need to maintain a positive relationship between children and the other parent while shielding them from conspiracy theories. Conflict frequently arose around medical decisions, with participants questioning the safety of leaving their child alone with the conspiracy‐believing parent. As one participant explained, “I am reluctant to let her go too long with him because I am afraid of a situation where she has some kind of medical need, and he doesn't get her appropriate attention. If something happened, would he take her to the hospital?” (Participant 9). Tension was further exacerbated by accusations from conspiracy‐believing ex‐partners, who often claimed poor parenting due to vaccination decisions or exposure to liberal views. Some children themselves became upset and chose to limit contact, adding further strain to co‐parenting and increasing stress for the participant. While many participants found relief after the breakup, some continued to face harassment and threats from their ex‐partners. Navigating life after separation thus involved both emotional recovery and ongoing practical challenges, especially when children were involved.

## DISCUSSION

This study explored how conspiracy beliefs shape and strain romantic relationships, addressing a gap in the qualitative research of the consequences of conspiracy beliefs, which has thus far focused on the effects of QAnon conspiracy beliefs on relationships generally – including family and friendships – rather than on romantic relationships specifically (Mastroni & Mooney, 2024; Waltman, 2023). This study focused on the experiences of romantic partners who did not share their partner's conspiracy beliefs and was also not restricted to the context of QAnon.

From these accounts, several key themes were developed, highlighting how conspiracy beliefs contributed to *relational strain, negative effects on participants, sense‐making, and seeking support and navigating the endings of the participants' relationships*. Participants described how conspiracy beliefs led to escalating arguments, emotional disconnection, and, in some cases, fear for their safety. Many experienced mental and physical health consequences, self‐doubt, and a sense of helplessness as their partner became increasingly immersed in conspiracy theories. Some felt as though they had ‘lost’ their partner entirely, describing them as a different person or a stranger due to the changes in their personality and worldview, while others attempted to make sense of their partner's beliefs, often viewing them as vulnerable to manipulation. For those with children, co‐parenting became a major concern, particularly regarding medical decisions. While some participants found support through social networks or professional help, many faced stigma and isolation. Ultimately, most relationships deteriorated, with many ending in separation, though some participants remained due to financial, emotional, or practical barriers.

The themes developed as part of this research show similarities to those found by Mastroni and Mooney (2024), but there were also some differences. For example, like Mastroni and Mooney, we found that conspiracy beliefs led to communication breakdown, emotional strain and relationship dissolution. Both studies also highlight how partners' conspiracy engagement led to escalating conflict and emotional disconnection, often leaving participants feeling as though they had ‘lost’ their partner. However, our study expands on this by emphasizing challenges with co‐parenting decisions, particularly regarding medical care, and by detailing how some participants viewed their partners as vulnerable to manipulation, rather than fully autonomous believers. Additionally, our findings provide further insight into the ways participants sought support, including turning to friends, online communities and professional help. In a minority of cases, our interviews provided insights for legal considerations through uncovering the alarming harassment that some interviewees experienced. Specifically, one participant reported that their partner received help from their fellow conspiracy believer community members to stalk and harass them.

### Theoretical implications

We now spend some time discussing each of the key themes identified in our study in more detail, relating each theme back to prior research on the psychology of conspiracy theories and research on close relationships.

#### Relational strain

In the early stages of their partner's conspiracy belief, participants reported repeated yet futile efforts in engaging with and debunking their views. The willingness to engage in this way despite repeated deadlock is mirrored by existing research suggesting that people are less likely to tolerate conspiracy beliefs in family relationships and are more likely to confront a family member (vs. co‐worker) who endorses them, particularly for conspiracy theories that have direct personal consequences (e.g., beliefs about vaccines, climate change and COVID‐19 safety measures; Okdie et al., 2023).

As conspiracy beliefs became more entrenched, participants described how their partner's worldview became all‐consuming and escalating arguments became common. Researchers have argued that when someone falls down the conspiracy “rabbit hole”, their beliefs accelerate over time, reinforcing a rigid belief system resistant to contradiction (Sutton & Douglas, 2022). Meta‐analytic evidence also suggests that the relationship between beliefs in conspiracy theories about COVID‐19 and their behavioural consequences becomes stronger over time (see Bierwiaczonek et al., 2020). Additionally, online echo chambers encourage conspiracy believers to disengage from non‐believers, instead surrounding themselves with like‐minded people (Brugnoli et al., 2019). This contributes to the reinforcement of their views and isolation from opposing perspectives. This may also reflect the tendency for conspiracy believers to report lower actively open‐minded thinking (see Bowes et al., 2023), a cognitive thinking style that, if neglected, can lead to the rejection of contradictory evidence. A qualitative study found that conspiracy believers often describe non‐believers as part of a passive, ignorant “herd”, making meaningful engagement with them difficult (Franks et al., 2017). As demonstrated in this study, conspiracy believers may become frustrated when others refuse to accept their worldview, while non‐believers grow frustrated from unsuccessful attempts to challenge these beliefs, leading to a cycle of avoidance and conflict.

Many participants experienced the *Psychosocial Death of their Partner and the Relationship* following their partner's ideological transformation (Sutton & Douglas, 2022). For those participants who additionally felt a lack of social support and empathy, the grief this caused became disenfranchised – meaning that the loss they felt because of the fundamental change in their partner was not openly acknowledged, validated, or mourned by society (Doka, 1989), leaving them unable to grieve in a socially legitimized way. Disenfranchised grief has typically been observed in the aftermath of, or in a relationship with, death such as for family members of individuals on death row (Jones & Beck, 2007) or families whose loved ones have died after police contact (Baker et al., 2019). However, non‐death or non‐physical losses, as in this case, may also trigger disenfranchised grief (Bailey, 2018; Blocker Turner & Stauffer, 2024). Crucially, disenfranchised grief can have detrimental consequences in that it not only exacerbates the trauma experienced by such losses, but stunts posttraumatic growth, leaving the individual unable to move on after suffering a loss (Doka, 2017; Spain et al., 2019).

In extreme cases, the psychosocial death of one's partner included them being perceived as dangerous. State anger has been found to mediate the relationship between conspiracy beliefs and the justification of violence (Jolley & Paterson, 2020), suggesting that conspiracy beliefs may exacerbate feelings of frustration and hostility, leading to more extreme responses. This is concerning given that conspiracy beliefs have also been linked to violent extremist intentions (Vegetti & Littvay, 2021), and that conspiracy believers are more likely to view violence as a legitimate means of achieving ideological goals (Rottweiler & Gill, 2020).

#### Negative effects on participants

Most participants reported that their partners' conspiracy beliefs took a toll not only on their relationship but also on their own psychological and physical health. Research on interpersonal conflict suggests that persistent, irresolvable disputes can have psychological and physiological consequences, including anxiety, depression and even a weakened immune system (see Kiecolt‐Glaser et al., 2010). The repeated nature of interpersonal conflicts surrounding conspiracy theories, especially within close relationships, appears to heighten participants' stress responses, contributing to both mental and physical strain. For those who had children with their partner, this was exacerbated by an added parental responsibility to ensure that their partner's beliefs do not harm their children, for instance, in medical situations. This aligns with research demonstrating the harmful consequences of conspiracy beliefs on health‐related outcomes (Jolley & Douglas, 2014a).

In addition to these health consequences, some participants described experiencing self‐doubt, questioning their own sanity because of their partner's unwavering certainty of conspiracy beliefs. Shared reality theory suggests that people derive a sense of reality and validation from those around them, particularly in close relationships (Echterhoff et al., 2009). If a partner holds drastically different views, this may disrupt this shared reality, leading to epistemic uncertainty and self‐questioning. Furthermore, research on attitude similarity shows that alignment in core beliefs (e.g., political ideology) plays a crucial role in maintaining relationship stability and self‐esteem (Huber & Malhotra, 2017). Indeed, partners who had once seemed aligned in values and interests now appeared like strangers, consumed by an alternate worldview that dominated their conversations and priorities. This shift in identity often led participants to question their relationship. Research on attitude similarity suggests that shared core beliefs are fundamental to relationship stability since alignment in values fosters mutual understanding and emotional closeness (Byrne et al., 1969; Montoya & Horton, 2012). Without this validation, participants described feeling isolated, with some struggling to trust their own perceptions.

Many participants also reported a growing sense of helplessness, feeling powerless to intervene or change their partner's beliefs. The concept of ‘rabbit hole syndrome’ describes how conspiracy beliefs deepen over time – as believers immerse themselves in conspiracy content, they isolate themselves from dissenting perspectives, making it increasingly difficult for close others to influence them (Sutton & Douglas, 2022). Once someone reaches this stage, external pressure or confrontation is likely to have limited effects and may not easily lead to change. Instead, belief entrenchment might occur, potentially further widening the divide between partners. In the current study, this sense of powerlessness and emotional exhaustion contributed to participants' overall distress, further eroding their well‐being and leaving them uncertain about the future of their relationship.

#### Sense‐making

Many participants described *becoming an expert* on conspiracy theories as they sought to understand their partner's beliefs and the broader conspiracy sphere. Research suggests that in response to stressful situations, some people are more likely to engage in information‐seeking behaviours to mitigate uncertainty (Miller, 1987). Participants in this study appeared to adopt this strategy, with some using it to anticipate and manage conflicts with their partner, while others saw it as a means to cope with the increasing conflicts in their relationship.

Some participants tried to make sense of and explain their partner's conspiracy beliefs by attributing them to external influences rather than a personal choice – for instance, by viewing their partner as *vulnerable to exploitation*. This may reflect attribution biases (Ross, 1977), in which people tend to explain others' behaviour through external forces rather than internal traits. Some participants framed their partners as passive victims of radicalization, comparing their experiences to cult indoctrination or psychological manipulation. Motivated reasoning (Kunda, 1990) may have shaped participants' views, as seeing their partner as a victim rather than an active believer may have helped them maintain their emotional connection. Exposure to conspiracy theories has also been shown to exacerbate feelings of victimhood (Bertin, 2024), suggesting that the partners' own victim perceptions may have influenced how participants viewed them.

In addition, some described conspiracy influencers as preying on vulnerable people for financial gain, reflecting the argument that ‘conspiracy entrepreneurs’ and influencers monetize distrust (Birchall, 2021). Rather than just a means to radicalize, participants believed their partners were targeted by people who mainly aimed to profit from their engagement. Some acknowledged that certain influencers may have political motives, but most emphasized financial exploitation, with their partners being drawn deeper into the conspiracy sphere through monetized content (Ballard et al., 2022). This demonstrates the unfortunate irony of falling into the conspiracy sphere: an overwhelming concern over uncovering conspiratorial plots is itself effectively a financial conspiracy to scam the followers of this movement.

#### Seeking support and navigating endings

Research suggests that social support plays a vital role in coping with relational stress and psychological distress (Cohen & Wills, 1985), and most of our respondents reported that they *found support* from others. In fact, scholars have argued that some individuals may similarly seek online conspiracist communities to achieve social support that they are unable to find elsewhere (see Biddlestone et al., 2021). However, as is often the case among conspiracy believers (Lantian et al., 2018), this was not always a successful strategy for participants. For some respondents, confiding in others came with worries of being stigmatized themselves or stigmatized because of their association with the conspiracy believer (i.e., stigma by association). This makes sense since conspiracy beliefs tend to be viewed as stigmatizing beliefs (Lantian et al., 2018). Some participants sought professional support to manage their stress and relational challenges. This type of support can be helpful, especially when dealing with an emotionally charged issue like a partner's belief system (Gergen et al., 2001).

Ultimately, most participants reported that their relationships deteriorated because of their partner's conspiracy beliefs. This typically led to the end of the relationship, which can result in feelings of grief, loss, but also relief (Amato, 2000). People often sought external support in leaving from their support network (family, friends and professionals), which aligns with existing research on the importance of social networks for helping individuals deal with the ending of a relationship (Canton, 2018). When support networks are strong, people are more likely to break free from damaging relationships (Rollè & Ramon, 2024). However, our participants sometimes experienced emotional and practical barriers in leaving their relationships. Feelings of guilt and potential harm to children, or concern for the partner's wellbeing, are well‐documented factors (e.g., Estrellado & Loh, 2013; Lindgren & Renck, 2009; Taherkhani et al., 2019). Practical matters such as financial responsibilities can further complicate matters, and people sometimes feel trapped by their life circumstances (Taherkhani et al., 2019). Once out of the relationship, however, participants often expressed relief and healing. This resonates with research suggesting that relationship disillusion, although emotionally challenging, can ultimately lead to greater personal growth and emotional recovery (Amato, 2000). For some participants, there were lingering effects such as issues with co‐parenting or manipulation from the ex‐partner, which corresponds with research on post‐relationship trauma and ongoing conflict, especially when the partner harasses the other party (e.g., Campbell et al., 2008; Postmus et al., 2011).

### Limitations and future directions

We need to note some limitations of the current research. Specifically, our sample was composed of people who responded to an advertisement about experiences of people who have had (or are having) relationships with conspiracy believers. It was a self‐selected group of individuals who wanted to talk about their concerns, and we therefore still know little about the experiences of people who are “suffering in silence”. On the other hand, people who have positive responses to their partner's adoption of conspiracy beliefs are unlikely to take part in research or seek help, potentially skewing our conclusions negatively. Self‐selection is a limitation of psychological research more generally but needs to be taken into consideration when making broader claims about the effects of conspiracy theories on people's relationships. In future research, this could be addressed by exploring the voices of those who maintain closeness and relationship satisfaction with conspiracy‐believing partners and understanding their experiences of relational negotiation, adaptation and resilience.

We also need to note some limitations of our research methodology. Although thematic analysis is advantageous in allowing researchers to examine the perspectives of different research participants, uncover similarities and differences, and reveal important insights, researchers are forced to take an epistemological position to underpin the study's claims, which introduces subjectivity and potential inconsistency to the analysis (Nowell et al., 2017). Nevertheless, we argue that the advantages of thematic analysis outweigh the costs in understanding the rich experiences of participants dealing with conspiracy theories in their close relationships.

Future research could focus on different questions, such as probing participants' personal wellbeing to attempt to understand how conspiracy theories are not only related to the psychological needs of the believer (Biddlestone et al., 2025; Douglas et al., 2017), but also the needs of the believer's partner. For example, in the current research, some participants appeared to engage in similar sense‐making processes to conspiracy believers. The difference here was that they sought to fully understand the phenomenon of conspiracy beliefs rather than seek evidence that supports their preconceived conspiracist conclusions. Given the variety of views endorsed by conspiracy believers, another avenue for future research may be to comparatively analyze different types or intensities of conspiracy belief (e.g., isolated vs. cross‐endorsed belief) to assess and isolate the conspiracy theories that seem to affect relationships the most. This could be done alongside research to understand how conspiracy theories affect different types of relationships (e.g., parent–child relationships, work relationships) and different relationship statuses (i.e., ongoing versus ended relationships) to examine whether those who remain in relationships – romantic or otherwise – with conspiracy believers adopt different coping strategies over time. Specifically, for instance, additional qualitative research could build upon our work by investigating the differences between those who remain in relationships with conspiracy believers and those who end such relationships and subsequently build a model of relationship dissolution.

Further studies on this topic could also consider in more detail how people engage with conspiracy believers in their close relationship circles. Researchers have started to consider some of the ways that people might engage with believers to change their minds (Douglas et al., 2024), and the current study provides some examples of such strategies. However, a more detailed qualitative examination of these strategies might help us understand how people can navigate conspiracy theories in their everyday lives, especially when the conspiracy theories can potentially influence important decisions such as those related to the health of themselves and their children.

### Conclusion

The current study examined the experiences of people who had been in or were currently in romantic relationships with conspiracy believers. We uncovered a range of negative and even potentially fatal consequences. We hope these findings can be used to illuminate why the concerns of those in close relationships with conspiracy believers should be taken seriously. We also hope that it can pave the way for theoretical models that account for these consequences. We recommend that further effort is put into providing therapeutic support for individuals in these circumstances. We also encourage the consideration of legal measures to address the potential dangers of conspiracy‐believing partners who engage in stalking behaviour and other abusive or coercive relationship dynamics.

## AUTHOR CONTRIBUTIONS

**Lea C. Kamitz:** Conceptualization; methodology; formal analysis; writing – review and editing; writing – original draft; investigation; data curation. **Ricky Green:** Conceptualization; writing – original draft; writing – review and editing; data curation. **Cassidy Rowden:** Project administration; data curation. **Daniel Toribio‐Flórez:** Writing – original draft; writing – review and editing. **Mikey Biddlestone:** Writing – original draft; writing – review and editing. **Karen M. Douglas:** Funding acquisition; writing – original draft; writing – review and editing; conceptualization; supervision.

## FUNDING INFORMATION

This research was supported by the European Research Council Advanced Grant “Consequences of conspiracy theories – CONSPIRACY_FX” Number: 101018262.

## CONFLICT OF INTEREST STATEMENT

We have no known conflict of interest to disclose.

## Data Availability

The data is not publicly available given its sensitive and potentially identifiable nature.
